# Supplementary material for: Chromosome segregation fidelity requires microtubule polyglutamylation by the cancer downregulated enzyme TTLL11
Source: Nat Commun. 2022 Nov 21;13:7147. doi: 10.1038/s41467-022-34909-y (PMC9681853; doi:10.1038/s41467-022-34909-y)
Supplement: Supplementary file 1 — Supplementary Information [file 41467_2022_34909_MOESM1_ESM.pdf]

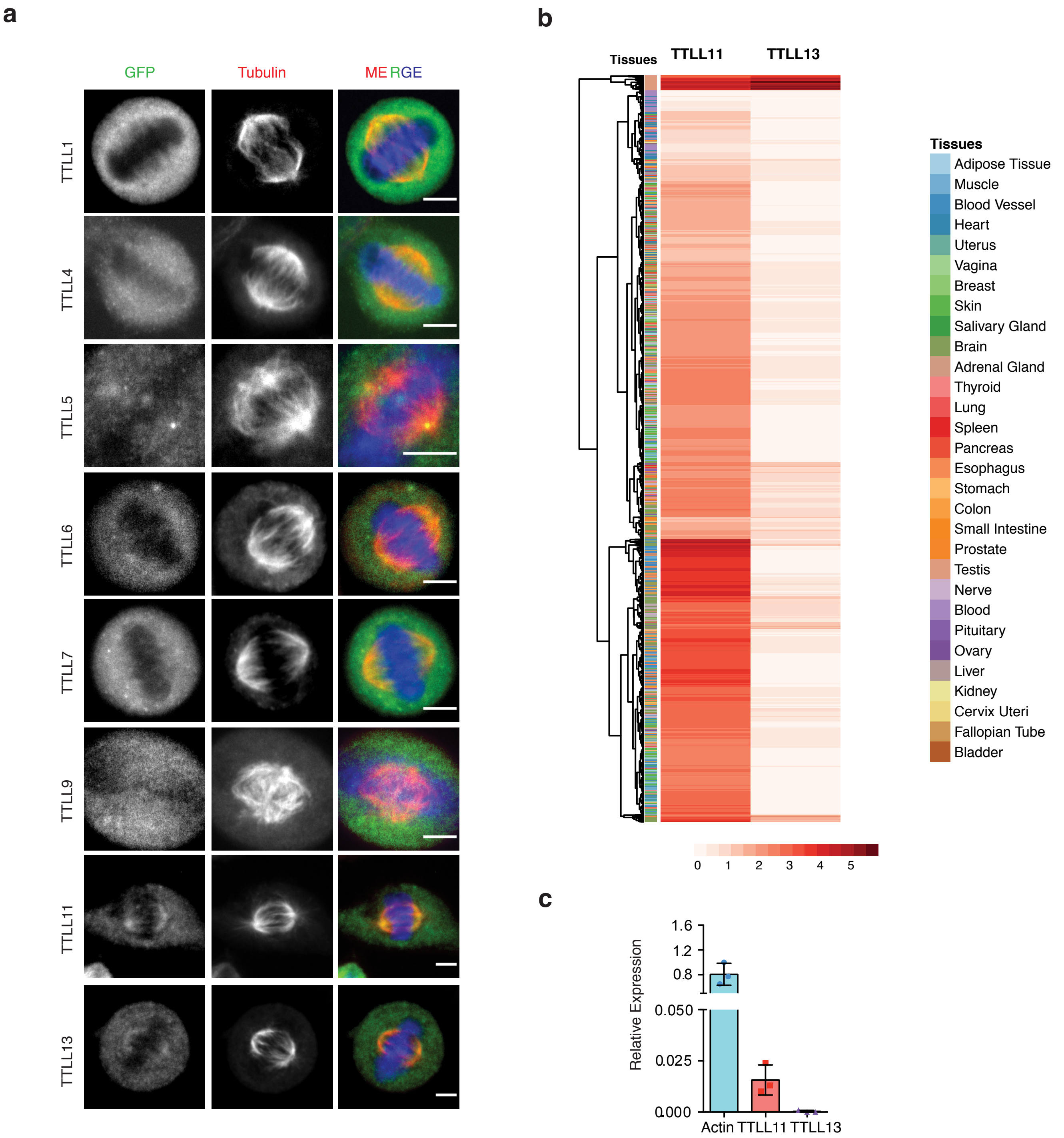

Supplementary Figure 1. TTLL11 and TTLL13 in human tissue and HeLa cells.
a- Fluorescent wide-field images of c-terminal GFP tagged TTLL (poly)glutamylases in mitotic HeLa cells, as indicated. Tubulin was stained with the DM1A antibody (red), GFP tagged TTLLs with anti-GFP (green), DNA was stained with DAPI (blue). Scale bars, 5 µm. Representative images of N = 2 independent experiments.
b- Expression levels of TTLL11 and TTLL13 across human tissues from GTEx illustrated as a red scale in log2(TPM+1).
c- Relative expression of actin, TTLL11 and TTLL13 in HeLa cells detected by RT-qPCR. The plot represent means from N = 3 independent experiments. Data are presented as mean values +/- SD.
Source data are provided as a Source Data file.

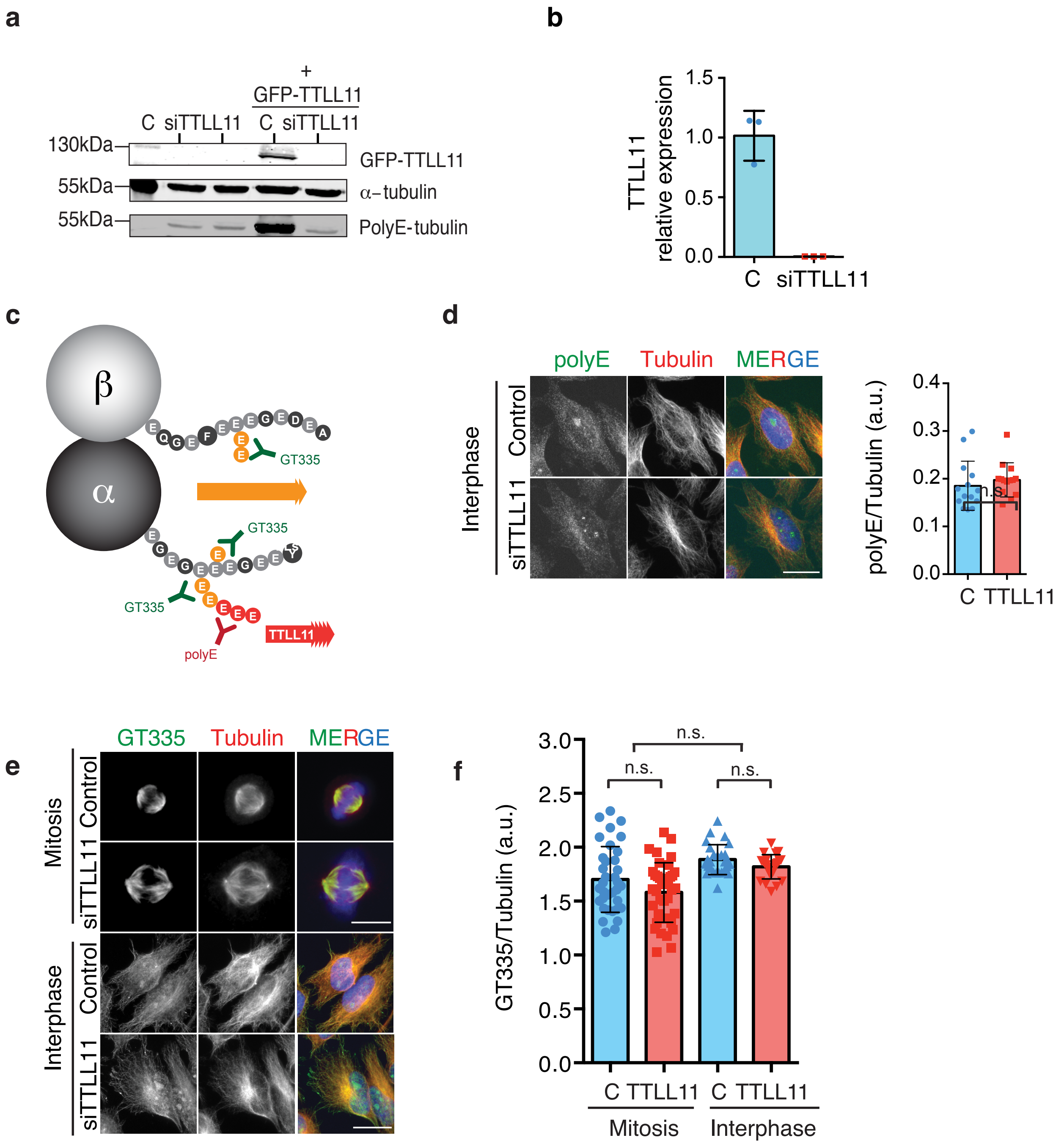

Supplementary Figure 2. TTLL11 silencing in HeLa cells and impact on MT (poly)glutamylation.
a- Western blot analysis of non-synchronized control and siTTLL11 HeLa cells expressing or not GFP-TTLL11. The blot was probed with an anti-GFP antibody to visualize exogenous GFP-TTLL11, the DM1a antibody loading control and the anti-PolyE antibody. Note the very low level of polyglutamylated MTs in control cells mostly in interphase and the increase levels in cells overexpressing GFP-TTLL11. Cell lysates were obtained 48 h after transfection. One representative WB of N = 2 independent experiments.
b- TTLL11 expression levels detected by RT-qPCR in control and TTLL11 silenced HeLa cells at 48 h after siRNA transfection. Expression levels represent means from N = 3 independent experiments. Data are presented as mean values +/- SD.
c- Schematic representation of the epitopes recognized by the GT335 and PolyE antibodies at the C-terminal tails of tubulin. GT335 specifically detects the glutamate branching out from alpha and beta tubulin C-terminus regions. Branching is catalysed by initiating TTLLs (orange). The polyE antibody detects linear glutamate chains of three or more glutamates that are catalysed by elongating polyglutamylases, such as TTLL11.
d- TTLL11 silencing has no impact on microtubule polyglutamylation levels detected with the PolyE antibody in interphase cells. Immunofluorescence images of control and siTTLL11 interphase HeLa cells, showing tubulin (red), polyE (green) and DAPI (blue). Scale bar, 10 µm. The graph shows the mean of the normalized anti-polyE antibody signal in control and siTTLL11 interphase cells. n(control) = 14; n(siTTLL11) = 14; N = 3 independent experiments. p-value is based on unpaired two-sided t-test with a 95% confidence interval. Data are presented as mean values +/- SD.
e- TTLL11 silencing has no impact on the levels of MT glutamylation detected by the GT335 antibody in interphase or mitotic HeLa cells. Immunofluorescence images of interphase and mitotic control and siTTLL11 HeLa cells, showing tubulin (red), GT335 (green) and DAPI (blue). Scale bars for cells in interphase, 20 µm; for cells in mitosis, 10 µm.
f- Graph showing the mean of normalized GT335 signal in the different conditions. Cells in mitosis: n(control) = 35, n(siTTLL11) = 38; cells in interphase, n (si-control) = 27 and n(siTTLL11) = 33; N = 2 independent experiments. p-values are based on one-way ANOVA test with a 95% confidence interval. Data are presented as mean values +/- SD.

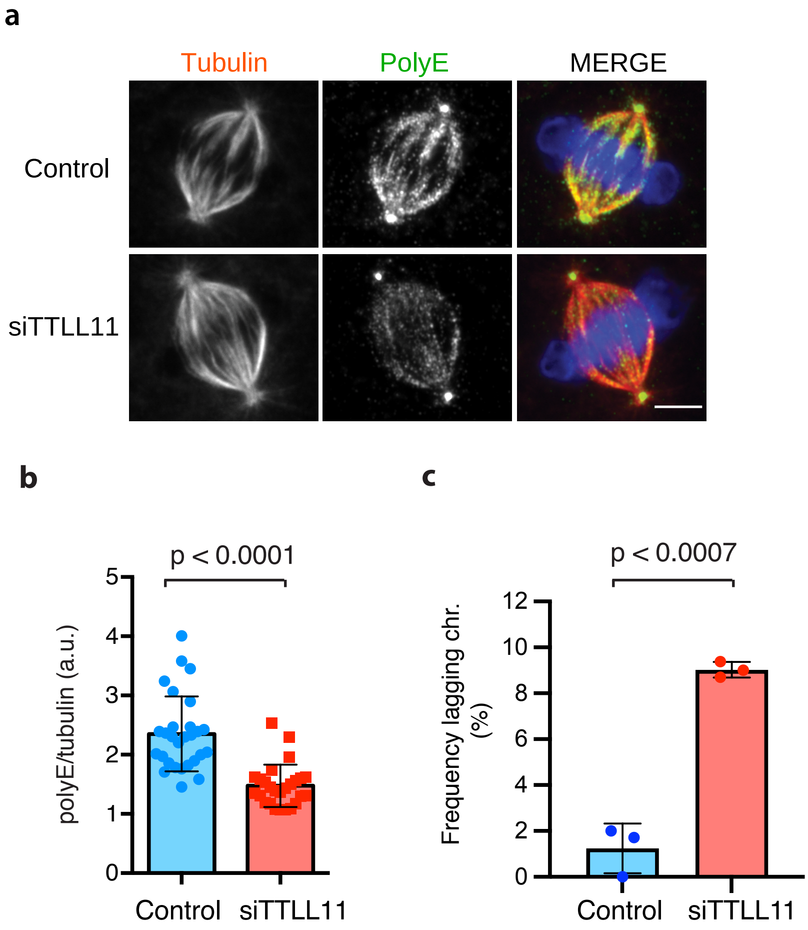

Supplementary Figure 3. TTLL11 silencing in hTERT-RPE1 cells impacts on MT (poly)glutamylation and lagging chromosomes frequency.
a- Immunofluorescence images of RPE1 metaphase spindles in control and siTTLL11 cells, showing PolyE (green), tubulin (red) and DNA (blue). Scale bar, 5 µm.
b- Graph showing the quantification of the polyE signal normalized to the total tubulin signal in control and siTTLL11 spindles and a graph showing the spindle length of RPE1 cells. n (control) = 28 cells and n (siTTLL11) > 30 cells. Graph representative of N = 3 independent experiments. The p-value is based on unpaired two-sided t-test with 95% confidence. Data are presented as mean values +/- SD.
c- Cumulative bar plot of RPE1 anaphase lagging chromosomes in control (n=205) and TTLL11 silenced cells (n=251), N=3 independent experiments. The p-value is based on χ^2 test with a 95% confidence interval. Bars represent the mean with error bars showing SD of three experiments.
Source data are provided as a Source Data file.

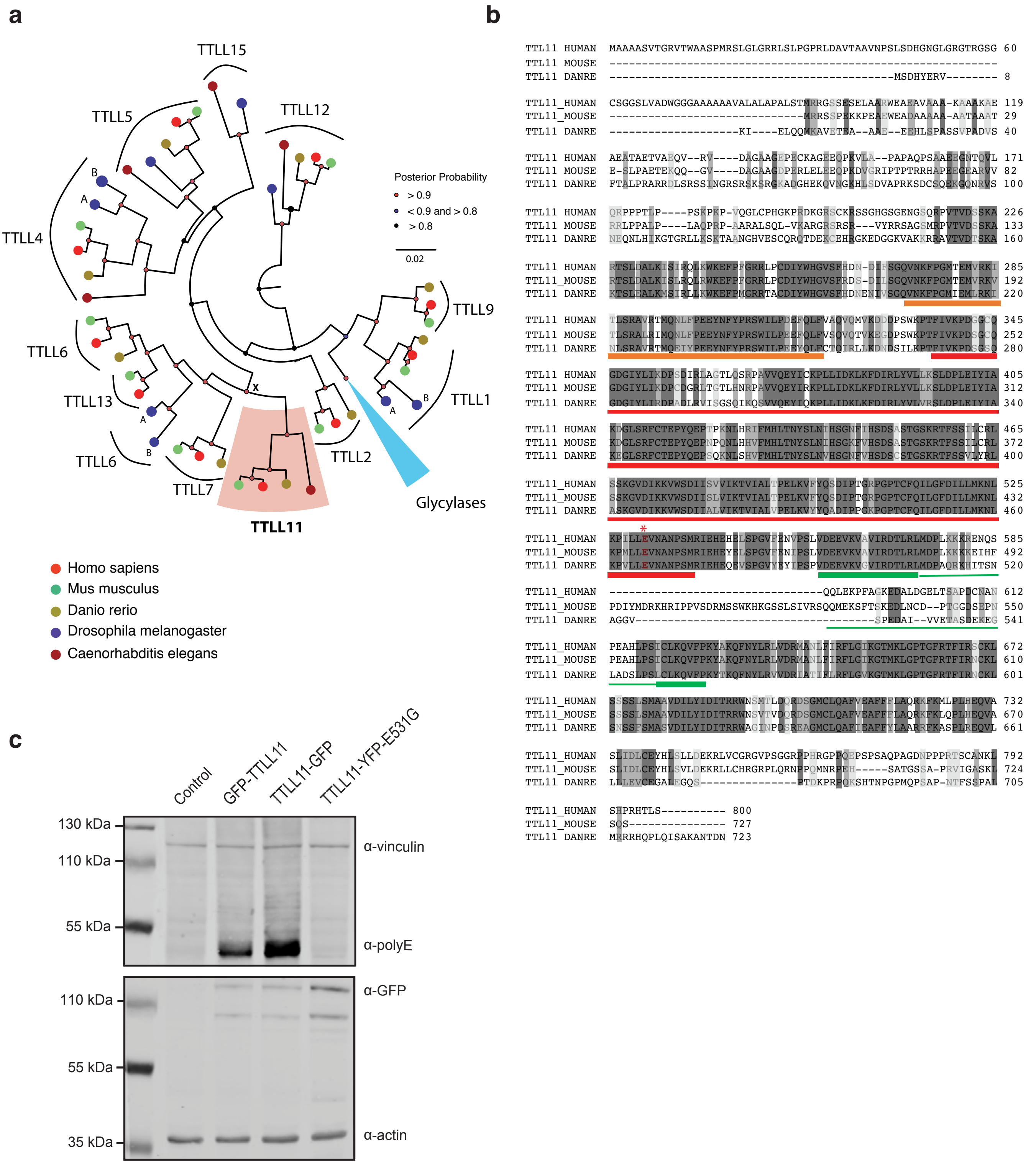

Supplementary Figure 4. Amino acid sequence analysis of TTLLs.

a- Phylogenetic tree of TTLLs enzymes. The tree includes all the TTLL enzymes from Homo sapiens, Mus musculus, Danio rerio, Drosophila melanogaster and Caenorhabditis elegans and was developed with posterior probability algorithm, setting TTLL12 as the outgroup. The TTLL11 branch is highlighted with a red shadow. The position of the glycylases is indicated with a light blue cone.

b- Protein sequence alignment of TTLL11 orthologues from human, mouse and zebrafish. TTLL11 protein sequences were aligned with the online tool Uniprot Align. Identical residues are highlighted in dark grey, and conserved changes and similarities, in light and very light grey, respectively. The colour bars below the alignment indicate the positions of the extended TTL domain (orange), the core TTL domain (red) (7) and the MT binding domain (green). The essential E466 required for catalytic activity that was mutated for rescue experiments is indicated in bold and an asterisk in red.

c- The substitution E531G abolishes TTLL11 catalytic activity. Western blot of lysates of asynchronous HEK293 cells expressing different fluorescently tagged TTLL11 proteins, as indicated. Both GFP-TTLL11 and TTLL11-GFP promote high levels of MT polyglutamylation whereas the catalytically dead variant TTLL11-E531G does not. MT polyglutamylation was monitored with the PolyE antibody that specifically recognizes stretches of three or more glutamates. An anti-GFP was used to monitor the level of expression of the recombinant proteins. Actin and Vinculin were used as loading controls.

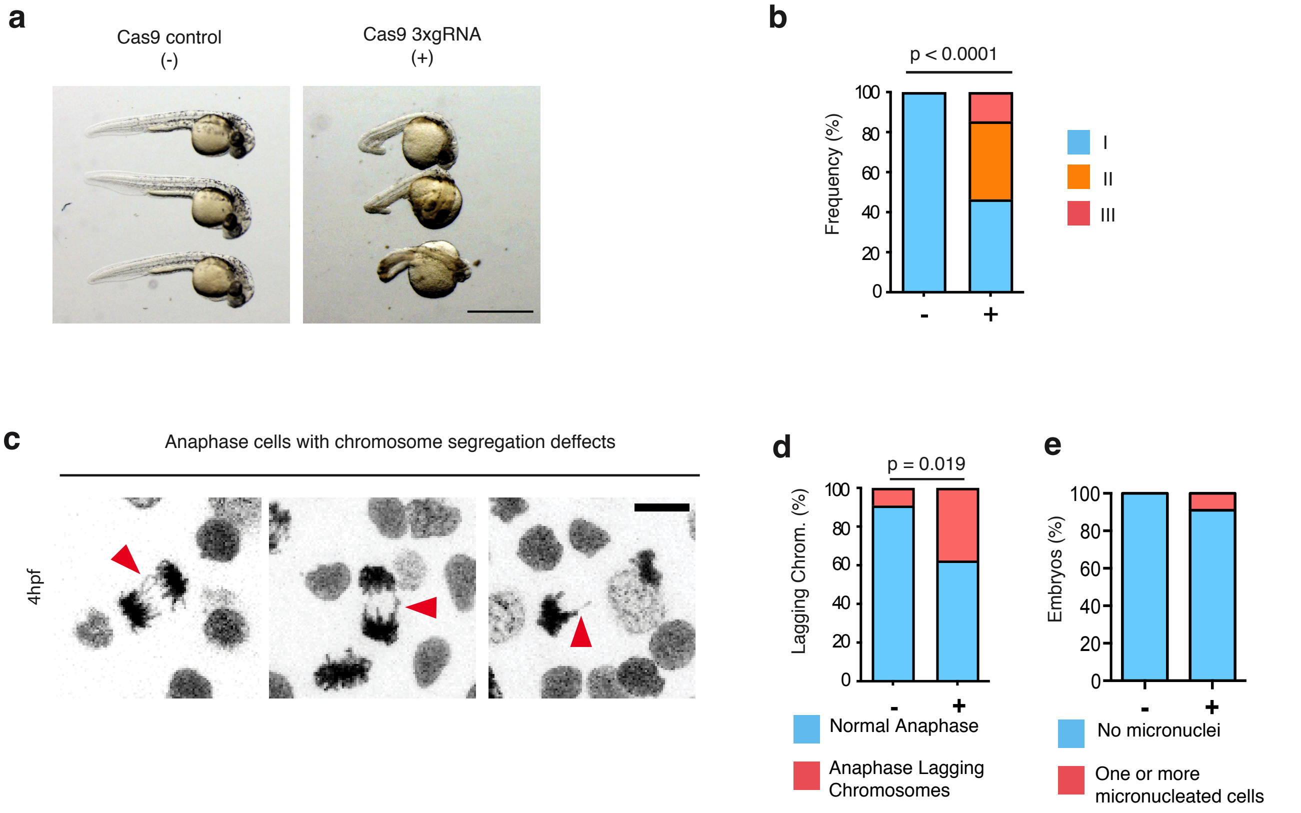

Supplementary Figure 5. Bi-allelic knockout of zfTTLL11.

a- WT zebrafish embryos (36 hpf) injected at the one cell stage with the three control gRNA and Cas9 as control (-) or the three gRNA against TTLL11 and Cas9 (Cas9 3xgRNA, +). Scale bar, 1 mm.

b- Cumulative bar plot of developmental defects in the two experimental conditions as described in a: severe (III); mild (II) or no defects (I) in 36-hpf embryos from N=2 independent experiments, control (-)(n =8 3) and Cas9 3xgRNA (+)(n = 83). p-values are based on a two-tailed 2 test with a 95% confidence interval.

c- Immunofluorescence images of zebrafish Cas9 3xgRNA injected embryos (4 hpf) showing chromosome segregation defects in anaphase cells. Scale bar, 10 μm.

d- Quantification of anaphase cells with lagging chromosomes in control (-) (n=24) and Cas9 3xgRNA (+)(n=32) injected embryos, N = 1. p-values values are based on a two-tailed χ2 test with a 95% confidence.

e- Quantification of fixed embryos showing at least one or more micronucleated cells in control (-) (n = 7) and Cas9 3xgRNA (+) (n = 11) embryos.

Source data are provided as a Source Data file.

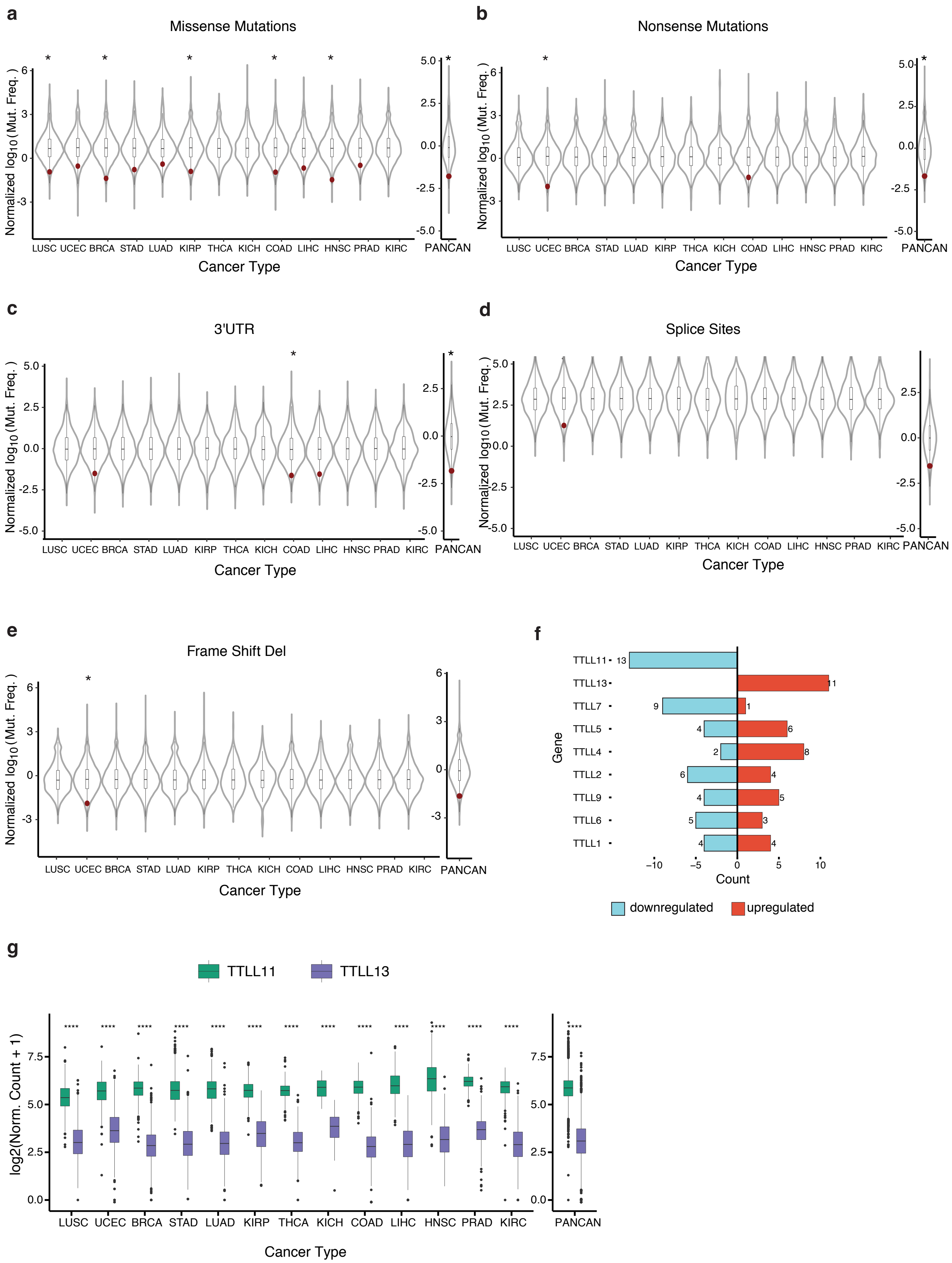
a
Missense Mutations
Normalized log10 (Mut. Freq.)
LUSC UCEC BRCA STAD LUAD KIRP THCA KICH COAD LIHC HNSC PRAD KIRC
PANCAN
Cancer Type
b
Nonsense Mutations
Normalized log10 (Mut. Freq.)
LUSC UCEC BRCA STAD LUAD KIRP THCA KICH COAD LIHC HNSC PRAD KIRC
PANCAN
Cancer Type
c
3'UTR
Normalized log10 (Mut. Freq.)
LUSC UCEC BRCA STAD LUAD KIRP THCA KICH COAD LIHC HNSC PRAD KIRC
PANCAN
Cancer Type
d
Splice Sites
Normalized log10 (Mut. Freq.)
LUSC UCEC BRCA STAD LUAD KIRP THCA KICH COAD LIHC HNSC PRAD KIRC
PANCAN
Cancer Type
e
Frame Shift Del
Normalized log10 (Mut. Freq.)
LUSC UCEC BRCA STAD LUAD KIRP THCA KICH COAD LIHC HNSC PRAD KIRC
PANCAN
Cancer Type
f
Gene
TTLL11
TTLL13
TTLL7
TTLL5
TTLL4
TTLL2
TTLL9
TTLL6
TTLL1
Count
downregulated
upregulated
g
TTLL11
TTLL13
log2(Norm. Count + 1)
LUSC UCEC BRCA STAD LUAD KIRP THCA KICH COAD LIHC HNSC PRAD KIRC
PANCAN
Cancer Type

Supplementary Figure 6. TTLL11 is rarely mutagenized in tumours.

a - e- TTLL11 is rarely mutagenized in tumours. Mutation frequencies per kilobase for every detected gene across 13 different cancer types. PANCAN represents the median mutation frequency per kilobase for every gene across cancers. TTLL11 is indicated in red. Mutation frequencies are classified according to their position or type of effect: (**a**) missense mutation, (**b**) nonsense mutation, (**c**) 3′-UTR, (**d**) splice sites and (**e**) frame shift deletion, Number of genes detected (see Supplementary Table 6). Nominal p-values: $*p < 0.05$; one-sided, one-sample Z tests.

f- TTLL11 is consistently downregulated in cancer. Graph showing the frequency of the differential expression and the direction (e.g., upregulated or downregulated) for each TTLL in primary tumours versus unmatched healthy solid tissue samples across 13 different types of cancer (two-sided Wilcoxon rank sum test; $FDR < 0.05$). The numbers indicate the number of types of cancer in which each TTLL is differentially expressed.

g- TTLL13 is less expressed than TTLL11 in cancer. Normalized expression of TTLL11 and TTLL13 in primary tumor samples across 13 different cancer types, separately and combined (PANCAN). Nominal p-values: $****p \leq 0.0001$; $***p \leq 0.001$; $**p \leq 0.01$; $*p \leq 0.05$; based on unmatched two-sided Wilcoxon rank sum tests. Number of samples by cancer type and gene (see Supplementary Table 7) In each box plot, the median value is indicated as a horizontal line and the lower and upper bounds of the box correspond to the first and third quartiles, respectively. The upper and lower whiskers range from the corresponding box hinges to the largest value no further than 1.5 times the inter-quartile range from the hinge. All outlying data points beyond the whiskers are plotted individually.

a

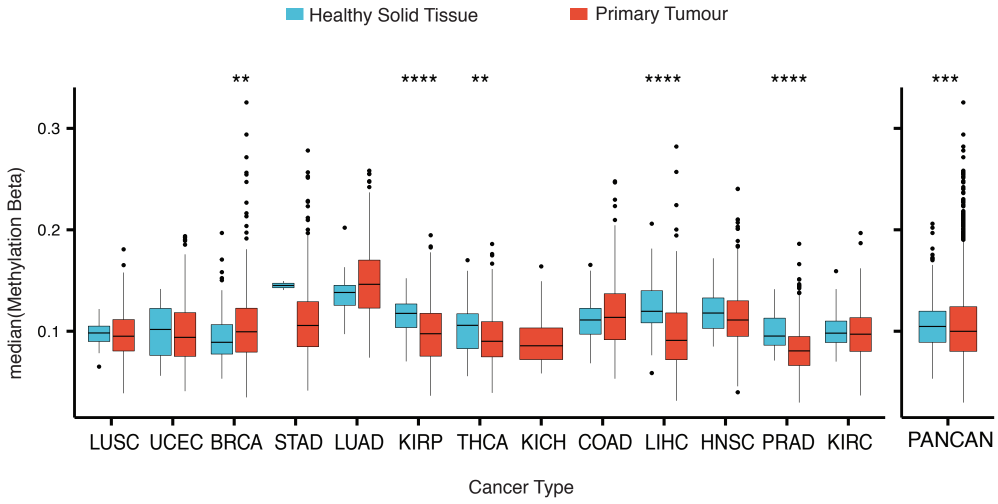

Supplementary Figure 7. Promoter methylation of TTLL11 does not explain the consistent transcriptomic downregulation of TTLL11 in tumours.
a- Median beta scores for all methylation sites within the 1,500bp upstream of TTLL11 transcription start site (y-axis) across 13 different cancer types (x-axis), separately and combined (PANCAN). Nominal p-values: (****) ≤ 0.0001, (***) ≤ 0.001, (**) ≤ 0.01, (*) ≤ 0.05, based on unmatched two-sided Wilcox Rank Sum tests. Numer of samples per cancer type and sample type (see Supplementary Table 8). In each box plot, the median value is indicated as a horizontal line and the lower and upper bounds of the box correspond to the first and third quartiles, respectively. The upper and lower whiskers range from the corresponding box hinges to the largest value no further than 1.5 times the inter-quartile range from the hinge. All outlying data points beyond the whiskers are plotted individually.

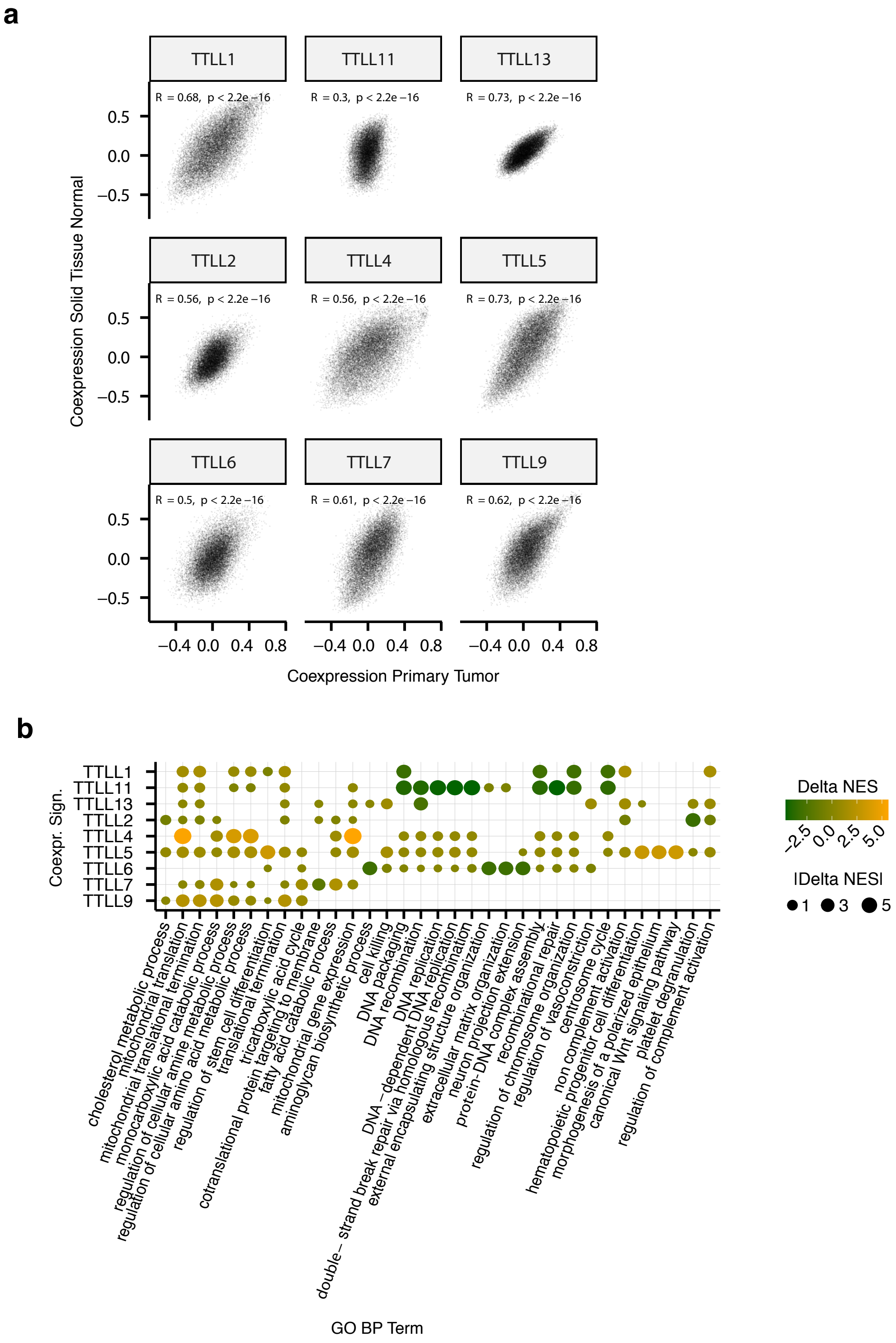

a
TTLL1
TTLL11
TTLL13
R = 0.68, p < 2.2e −16
R = 0.3, p < 2.2e −16
R = 0.73, p < 2.2e −16
TTLL2
TTLL4
TTLL5
R = 0.56, p < 2.2e −16
R = 0.56, p < 2.2e −16
R = 0.73, p < 2.2e −16
TTLL6
TTLL7
TTLL9
R = 0.5, p < 2.2e −16
R = 0.61, p < 2.2e −16
R = 0.62, p < 2.2e −16
0.5
0.0
−0.5
−0.4 0.0 0.4 0.8
Coexpression Solid Tissue Normal
Coexpression Primary Tumor
b
Coexpr. Sign.
TTLL1
TTLL11
TTLL13
TTLL2
TTLL4
TTLL5
TTLL6
TTLL7
TTLL9
cholesterol metabolic process
mitochondrial translation
mitochondrial translational termination
monocarboxylic acid catabolic process
regulation of cellular amine metabolic process
regulation of cellular amino acid metabolic process
regulation of stem cell differentiation
translational termination
tricarboxylic acid cycle
cotranslational protein targeting to membrane
fatty acid catabolic process
mitochondrial gene expression
aminoglycan biosynthetic process
cell killing
DNA packaging
DNA recombination
DNA replication
DNA−dependent DNA replication
double− strand break repair via homologous recombination
external encapsulating structure organization
extracellular matrix organization
neuron projection extension
protein-DNA complex assembly
recombinational repair
regulation of chromosome organization
regulation of vasoconstriction
centrosome cycle
non complement activation
hematopoietic progenitor cell differentiation
morphogenesis of a polarized epithelium
canonical Wnt signaling pathway
platelet degranulation
regulation of complement activation
GO BP Term
Delta NES
−2.5
0.0
2.5
5.0
|Delta NES|
1
3
5

c

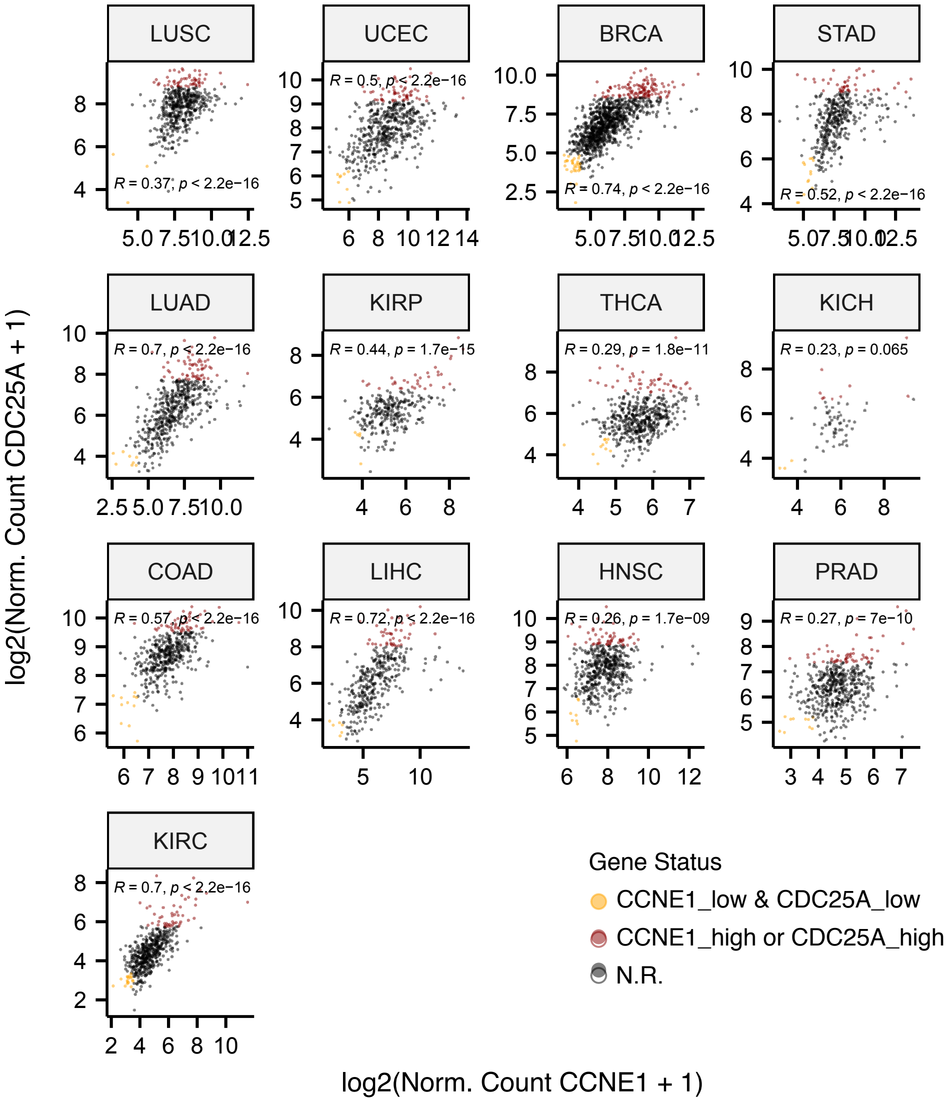

Supplementary Figure 8. Coexpression-based enrichment analysis of TTLL glutamylases and putative upstream regulators of TTLL11.

a- Co-expression signature between each TTLL glutamylase and the rest of the genes in samples from primary tumours (x-axis) and healthy solid tissue (y-axis). In each subpanel, Spearman correlations between the two signatures and corresponding nominal p-values are shown.

b- Differences in normalized enrichment scores (NES) (Delta NES) between the tumour and healthy solid tissue coexpression signatures (x-axis) of each TTLL glutamylase enzyme correlated against the rest of the genes. NES were obtained through gene-set enrichment analysis (GSEA) and show whether a coexpression signature is significantly enriched (FDR < 0.05) in genes from a biological process listed in the Gene Ontology database.

c- Normalized expression of genes CCNE1 (x-axis) and CDC25A (y-axis) in primary tumor samples across 13 different human cancer types (as indicated at the top of each graph). The dot colours indicate high expression levels of either CCNE1 or CDC25A ("CCNE1_high & CDC25A_high", red), low levels of both ("CCNE_low & CDC25A_low", yellow), or was not relevant for this classification ("N.R.", black). In each subpanel, Spearman correlations between the two signatures and corresponding p-values are shown.

Supplementary Tables

Table 1. TTLL11 differential expression in tumours (Fig. 4A)

log_2(Norm.Count+1)

cancer_type	sample_type	n	mean	SD
LUSC	Solid Tissue Normal	51	6,375	0,311
LUSC	Primary Tumour	502	5,349	0,713
UCEC	Solid Tissue Normal	34	6,513	0,441
UCEC	Primary Tumour	532	5,672	0,701
BRCA	Solid Tissue Normal	114	6,580	0,356
BRCA	Primary Tumour	1097	5,816	0,555
STAD	Solid Tissue Normal	35	6,569	0,969
STAD	Primary Tumour	415	5,813	0,789
LUAD	Solid Tissue Normal	59	6,326	0,327
LUAD	Primary Tumour	515	5,745	0,751
KIRP	Solid Tissue Normal	32	6,079	0,304
KIRP	Primary Tumour	290	5,709	0,549
THCA	Solid Tissue Normal	59	6,005	0,278
THCA	Primary Tumour	505	5,714	0,427
KICH	Solid Tissue Normal	25	6,238	0,352
KICH	Primary Tumour	66	5,825	0,551
COAD	Solid Tissue Normal	41	6,365	0,633
COAD	Primary Tumour	452	5,902	0,491
LIHC	Solid Tissue Normal	50	6,221	0,443
LIHC	Primary Tumour	371	5,997	0,693
HNSC	Solid Tissue Normal	44	6,739	0,629
HNSC	Primary Tumour	520	6,339	0,953
PRAD	Solid Tissue Normal	52	6,468	0,448
PRAD	Primary Tumour	497	6,212	0,394
KIRC	Solid Tissue Normal	72	6,084	0,283
KIRC	Primary Tumour	533	5,863	0,566

Abbreviation legend: https://gdc.cancer.gov/node/677

Table 2. Primers list

Target	sense	sequence
TTLL11	Fw	5'—ACTTCTACCCTCGCTCATGG—3'
	Rv	5'—CCTGACAACCACCATCAGGTT—3'
TTLL13	Fw	5'—ACCAACCCCTCTAACTCTTC—3'
	Rv	5'—TTTCCGCCTTCTCTTCCTC—3'
Actin	Fw	5'—CGAGAAGATGACCCAGATCATG—3'
	Rv	5'—CCACAGGACTCCATGCCCAGG—3'

zfTTLL11	Fw	5'—GTGGACATCAAGAAGGTCTG—3'
	Rv	5'—AAAGTCTAGGACCCGAAAC—3'

Table 3. n number Figure 4a

Number of samples per cancer type and sample type
LUSCSTN (n=51)
LUSCPT (n=502)
UCECSTN (n=34)
UCECPT (n=532)
BRCASTN (n=114)
BRCAPT (n=1097)
STADSTN (n=35)
STADPT (n=415)
LUADSTN (n=59)
LUADPT (n=515)
KIRPSTN (n=32)
KIRPPT (n=290)
THCASTN (n=59)
THCAPT (n=505)
KICHSTN (n=25)
KICHPT (n=66)
COADSTN (n=41)
COADPT (n=452)
LIHCSTN (n=50)
LIHCPT (n=371)
HNSCSTN (n=44)
HNSCPT (n=520)
PRADSTN (n=52)
PRADPT (n=497)
KIRCSTN (n=72)
KIRCPT (n=533).

Table 4. n number Figure 4b

Number of genes detected per cancer type:
LUSC (n=20243)
UCEC (n=17507)
BRCA (n=20248)
STAD (n=16765)
LUAD (n=20190)
KIRP (n=20204)
THCA (n=20151)
KICH (n=19777)
COAD (n=17507)
LIHC (n=20152)
HNSC (n=20260)
PRAD (n=19088)
KIRC (n=20243)
PANCAN (n=20294).

Table 5. n number Figure 6e

Number of samples per cancer type and sample type
LUSCSTN (n=51)
LUSCCCNE1_high or CDC25A_high (n=56)
LUSCCCNE1_low & CDC25A_low (n=3)
UCECSTN (n=34)
UCECCCNE1_high or CDC25A_high (n=57)
UCECCCNE1_low & CDC25A_low (n=9)
BRCASTN (n=114)
BRCACCNE1_high or CDC25A_high (n=121)
BRCACCNE1_low & CDC25A_low (n=31)
STADSTN (n=35)
STADCCNE1_high or CDC25A_high (n=44)
STADCCNE1_low & CDC25A_low (n=13)
LUADSTN (n=59)
LUADCCNE1_high or CDC25A_high (n=58)
LUADCCNE1_low & CDC25A_low (n=8)
KIRPSTN (n=32)
KIRPCCNE1_high or CDC25A_high (n=33)
KIRPCCNE1_low & CDC25A_low (n=5)
THCASTN (n=59)
THCACCNE1_high or CDC25A_high (n=46)
THCACCNE1_low & CDC25A_low (n=11)
KICHSTN (n=25)
KICHCCNE1_high or CDC25A_high (n=9)
KICHCCNE1_low & CDC25A_low (n=3)
COADSTN (n=41)
COADCCNE1_high or CDC25A_high (n=49)

COADCCNE1_low & CDC25A_low (n=11)
LIHCSTN (n=50)
LIHCCCNE1_high or CDC25A_high (n=42)
LIHCCCNE1_low & CDC25A_low (n=6)
HNSCSTN (n=44)
HNSCCCNE1_high or CDC25A_high (n=54)
HNSCCCNE1_low & CDC25A_low (n=8)
PRADCCNE1_high or CDC25A_high (n=54)
PRADCCNE1_low & CDC25A_low (n=10)
KIRCSTN (n=72)
KIRCCCNE1_high or CDC25A_high (n=47)
KIRCCCNE1_low & CDC25A_low (n=17)
PANCANSTN (n=668)
PANCANCCNE1_high or CDC25A_high (n=670)
PANCANCCNE1_low & CDC25A_low (n=135).

Table 6. n number Supplementary Figure 6a-e

missense mutation	nonsense mutation;	3'-UTR;	splice sites;	frame shift deletion.
LUSC (n=16220)	LUSC (n=5063);	LUSC (n=4919);	LUSC (n=2513);	LUSC (n=2503);
UCEC (n=18584)	UCEC (n=11615);	UCEC (n=14970);	UCEC (n=5880);	UCEC (n=6926);
BRCA (n=14008)	BRCA (n=2827);	BRCA (n=4820);	BRCA (n=1194);	BRCA (n=5666);
STAD (n=16815)	STAD (n=4369);	STAD (n=5922);	STAD (n=2210);	STAD (n=7421);
LUAD (n=16275)	LUAD (n=5632);	LUAD (n=4636);	LUAD (n=2867);	LUAD (n=2767);
KIRP (n=7652)	KIRP (n=702);	KIRP (n=1495);	KIRP (n=415);	KIRP (n=1319);
THCA (n=3391)	THCA (n=263);	THCA (n=301);	THCA (n=111);	THCA (n=158);
KICH (n=1616)	KICH (n=122);	KICH (n=190);	KICH (n=50);	KICH (n=53);
COAD (n=17074)	COAD (n=5286);	COAD (n=7539);	COAD (n=2102);	COAD (n=6184);
LIHC (n=12017)	LIHC (n=1634);	LIHC (n=3212);	LIHC (n=1028);	LIHC (n=1110);

HNSC (n=14904) PRAD (n=9302) KIRC (n=8791) PANCAN (n=18807)	HNSC (n=3375); PRAD (n=1011); KIRC (n=930); PANCAN (n=15455));	HNSC (n=3454); PRAD (n=1026); KIRC (n=1479); PANCAN (n=16552);	HNSC (n=1293); PRAD (n=391); KIRC (n=548); PANCAN (n=10370))	HNSC (n=1538); PRAD (n=673); KIRC (n=1416); PANCAN (n=14453)).

Table 7. n number Supplementary Figure 6g

Number of samples by cancer type and gene:
LUSCTTLL11 (n=506)<br>LUSCTTLL13 (n=506)<br>UCECTTLL11 (n=547)<br>UCECTTLL13 (n=547)<br>BRCATTLL11 (n=1101)<br>BRCATTLL13 (n=1101)<br>STADTTLL11 (n=478)<br>STADTTLL13 (n=478)<br>LUADTTLL11 (n=520)<br>LUADTTLL13 (n=520)<br>KIRPTTLL11 (n=291)<br>KIRPTTLL13 (n=291)<br>THCATTLL11 (n=507)<br>THCATTLL13 (n=507)<br>KICHTTLL11 (n=66)<br>KICHTTLL13 (n=66)<br>COADTTLL11 (n=461)<br>COADTTLL13 (n=461)<br>LIHCTTLL11 (n=377)<br>LIHCTTLL13 (n=377)<br>HNSCTTLL11 (n=528)<br>HNSCTTLL13 (n=528)<br>PRADTTLL11 (n=498)<br>PRADTTLL13 (n=498)<br>KIRCTTLL11 (n=536)<br>KIRCTTLL13 (n=536)<br>PANCANTTLL11 (n=6416)<br>PANCANTTLL13 (n=6416).

Table 8. n number Supplementary Figure 7a

Numer of samples per cancer type and sample type:
LUSCSTN (n=41)
LUSCPT (n=364)
UCECSTN (n=45)
UCECPT (n=418)
BRCASTN (n=87)
BRCAPT (n=779)
STADSTN (n=2)
STADPT (n=393)
LUADSTN (n=30)
LUADPT (n=456)
KIRPSTN (n=43)
KIRPPT (n=271)
THCASTN (n=56)
THCAPT (n=503)
KICHPT (n=65)
COADSTN (n=34)
COADPT (n=288)
LIHCSTN (n=50)
LIHCPT (n=374)
HNSCSTN (n=50)
HNSCPT (n=523)
PRADSTN (n=50)
PRADPT (n=495)
KIRCSTN (n=157)
KIRCPT (n=312)
PANCANSTN (n=645)
PANCANPT (n=5241)

Table 9

crRNA name	Location	Target sequence [PAM]	GeneScan forward primer	GeneScan reverse primer
ttll11-1	Exon 2	GCCTGCGATATCTACTGGCA**[CGG]**	TGTAAAACGACGGCCAGT TCAGATGCGAGTTCTTGTGTTT	ATTTGTTGACTTGCCCAGAGAC
ttll11-3	Exon 3	TCGAGCTGTGCGCACCATGC**[AGG]**	TGTAAAACGACGGCCAGT TGACTCCCCTGTAGGGCTATAA	TGTGTGCAGAAGAGCTGGTATT
ttll11-4	Exon 4	GAGAACCTGAGATCACCCGA**[AGG]**	TGTAAAACGACGGCCAGT TAACGATTCCATTCTCAAACCC	GAACCTGGACAAGCCTTCTTTA

Supplementary methods

Publically available datasets used

Pan-cancer normalized gene expression, methylation and somatic mutations, aneuploidy scores and gene length were obtained from the TCGA Consortium. Phylogeny data were retrieved from ensembl (www.ensembl.org). The following publically available datasets were used: GTEx [https://www.gtexportal.org/home/datasets#filesetFilesDiv13] Consortium (v8).

Gene mRNA levels as TPM (“*GTEx_Analysis_2017-06-05_v8_RNASeQCv1.1.9_gene_tpm.gct.gz*”) and corresponding sample metadata (“*GTEx_Analysis_v8_Annotations_SampleAttributesDS.txt*”).

TCGA Consortium Data available at UCSC’s XenaBrowser [https://xenabrowser.net/datapages/?cohort=TCGA%20Pan-Cancer%20(PANCAN)&removeHub=https%3A%2F%2Fxena.treehouse.gi.ucsc.edu%3A443]. Gene mRNA levels as pan-cancer normalized counts (“*Batch effects normalized mRNA data*”), somatic mutations (“*MC3 public version*”), and DNA methylation (“*DNA methylation (Methylation250K)*”).

TCGA Aneuploidy Scores obtained from Taylor et al. Supplementary Table 2.
Taylor AM, Shih J, Ha G, Gao GF, Zhang X, Berger AC, Schumacher SE, Wang C, Hu H, Liu J, Lazar AJ; Cancer Genome Atlas Research Network, Cherniack AD, Beroukhim R, Meyerson M. Genomic and Functional Approaches to Understanding Cancer Aneuploidy. Cancer Cell. 2018 Apr 9;33(4):676-689.e3. DOI: https://doi.org/10.1016%2Fj.ccell.2018.03.007.

RNA sequencing upon overexpression of oncogenes in cancer cell lines.
NCBI Gene Expression Omnibus ID GSE185512. Next generation sequencing facilitates quantitative analysis of oncogene induced transcriptomes.
https://www.ncbi.nlm.nih.gov/geo/query/acc.cgi?acc=GSE185512
Guerrero Llobet, S. *et al.* An mRNA expression-based signature for oncogene-induced

replication-stress. *Oncogene* **41**, 1216-1224 (2022).

Gene sets of transcription factor targets.

Lachmann A, Xu H, Krishnan J, Berger SI, Mazloom AR, Ma'ayan A. ChEA: transcription factor regulation inferred from integrating genome-wide ChIP-X experiments. Bioinformatics. 2010 Oct 1;26(19):2438-44. DOI: https://doi.org/10.1093/bioinformatics/btq466.

https://maayanlab.cloud/Harmonizome/dataset/CHEA+Transcription+Factor+Targets

List of software used for data analysis

software	version
snakemake	5.31.1
gtftools	0.8.5
Python	3.8.3
pandas	1.3.0
R	4.1.0
clusterProfiler	4.0.5
ComplexHeatmap	2.9.3
cowplot	1.1.1
doParallel	1.0.16
enrichplot	1.12.2
extrafont	0.17
GeneBreak	1.22.0
GenomicRanges	1.44.0
GEOquery	2.60.0
ggplotify	0.1.0
ggpubr	0.4.0

ggrepel	0.9.1
ggvenn	0.1.9
gtools	3.9.2
latex2exp	0.5.0
limma	3.48.3
magrittr	2.0.3
optparse	1.7.1
org.Hs.eg.db	3.13.0
patchwork	1.1.1
pheatmap	1.0.12
readxl	1.3.1
reshape2	1.4.4
scattermore	0.7
tidyverse	1.3.1
writexl	1.4.0
