# Supplementary material for: Chromosome segregation fidelity requires microtubule polyglutamylation by the cancer downregulated enzyme TTLL11
Source: Nat Commun. 2022 Nov 21;13:7147. doi: 10.1038/s41467-022-34909-y (PMC9681853; doi:10.1038/s41467-022-34909-y)
Supplement: Supplementary file 3 — Description of Additional Supplementary Files [file 41467_2022_34909_MOESM3_ESM.pdf]

Description of Additional Supplementary Files

File Name: Supplementary Movie 1
Description: **Control and siTTLL11 Hela cells show no differences in mitotic progression.** Spinning-disk confocal time-lapse imaging of HeLa Kyoto cells stably expressing H2B–mRFP and α-tubulin-GFP under the indicated experimental conditions. Images were recorded every 2 min and 10 s. Time = hh:mm:ss. Scale bar, 20 μm.

File Name: Supplementary Movie 2
Description: **zfTTLL11 localizes to the mitotic spindle in zebrafish embryos.** Confocal time-lapse imaging of zebrafish embryo Tg(bactin:H2AmCherry) strain microinjected with GFP-zfTTLL11 under the indicated experimental conditions. Images were recorded every 45 s. Time = mm:ss. Scale bar, 10 μm.

File Name: Supplementary Movie 3
Description: **MO injected embryos display high frequency of micronuclei, some arising from anaphase lagging chromosomes.** Confocal time-lapse imaging of zebrafish embryo Tg(bactin:H2AmCherry) strain microinjected with GFP-zfTTLL11 under the indicated experimental conditions. Images were recorded every 45 s. Time = mm:ss. Scale bar, 20 μm.

File Name: Supplementary Movie 4
Description: **MO injected embryos show impaired chromosome segregation during anaphase.** Confocal time-lapse imaging of zebrafish embryo Tg(bactin:H2AmCherry) strain microinjected with GFP-zfTTLL11 under the indicated experimental conditions. Images were recorded every 45 s. Time = mm:ss. Scale bar, 20 μm.

File Name: Supplementary Movie 5
Description: **The SAC is not weakened by TTLL11 silencing.** Spinning-disk confocal time lapse imaging of HeLa Kyoto cells stably expressing H2B–mRFP and α-tubulin-GFP under the indicated experimental conditions. Images were recorded every 2 min and 10 s. Time = hh:mm:ss. Scale bar, 10 μm.

File Name: Supplementary Movie 6
Description: **Spindle MT poleward flux is impaired by siTTLL11 silencing in HeLa cells.** Confocal time-lapse imaging of HeLa cells stably expressing H2B–mRFP and PA-α-tubulin–GFP under the indicated experimental condition. Images were recorded every 10 s. Time = mm:ss. Scale bar, 5 μm.
