# Supplementary material for: Chromosome segregation fidelity requires microtubule polyglutamylation by the cancer downregulated enzyme TTLL11
Source: Nat Commun. 2022 Nov 21;13:7147. doi: 10.1038/s41467-022-34909-y (PMC9681853; doi:10.1038/s41467-022-34909-y)
Supplement: Supplementary file 10 — Reporting Summary [file 41467_2022_34909_MOESM10_ESM.pdf]

nature portfolio

Corresponding author(s): Isabelle Vernos

Last updated by author(s): Nov 8, 2022

Reporting Summary

Nature Portfolio wishes to improve the reproducibility of the work that we publish. This form provides structure for consistency and transparency in reporting. For further information on Nature Portfolio policies, see our Editorial Policies and the Editorial Policy Checklist.

Statistics

For all statistical analyses, confirm that the following items are present in the figure legend, table legend, main text, or Methods section.

n/a	Confirmed	
☐	☒	The exact sample size (*n*) for each experimental group/condition, given as a discrete number and unit of measurement
☐	☒	A statement on whether measurements were taken from distinct samples or whether the same sample was measured repeatedly
☐	☒	The statistical test(s) used AND whether they are one- or two-sided<br>*Only common tests should be described solely by name; describe more complex techniques in the Methods section.*
☒	☐	A description of all covariates tested
☒	☐	A description of any assumptions or corrections, such as tests of normality and adjustment for multiple comparisons
☐	☒	A full description of the statistical parameters including central tendency (e.g. means) or other basic estimates (e.g. regression coefficient) AND variation (e.g. standard deviation) or associated estimates of uncertainty (e.g. confidence intervals)
☐	☒	For null hypothesis testing, the test statistic (e.g. *F*, *t*, *r*) with confidence intervals, effect sizes, degrees of freedom and *P* value noted<br>*Give P values as exact values whenever suitable.*
☒	☐	For Bayesian analysis, information on the choice of priors and Markov chain Monte Carlo settings
☒	☐	For hierarchical and complex designs, identification of the appropriate level for tests and full reporting of outcomes
☒	☐	Estimates of effect sizes (e.g. Cohen's *d*, Pearson's *r*), indicating how they were calculated

Our web collection on statistics for biologists contains articles on many of the points above.

Software and code

Policy information about availability of computer code

Data collection

The XenaBrowser was used to obtain publicly available data on pan-cancer normalized gene expression, copy number variation, and somatic mutations in patients from The Cancer Genome Atlas (TCGA). The aneuploidy scores were obtained directly from Taylor et al. (2018). Gene lengths were obtained from the latest human genome reference (GRCh38) made available through the object “ens.gene.ann.hg38” in the R package GeneBreak. The full data collection and analysis pipeline is available at https://github.com/MiqG/Zadra_2021.

Data analysis

Microscopy:
-Andoriq 3
-LAS X 4.13
We used the following open source packages in R programming language:
- tidyverse 1.3.1
- limma 3.48.3
- doParallel 1.0.16
- GeneBreak 1.22.0
- reshape2 1.4.4
- latex2exp 0.5.0
- gtools 3.9.2
- writexl 1.4.0
- maggritr 2.0.1
- ggpubr 0.4.0
- pheatmap 1.0.12
- ggrepel 0.9.1
- patchwork 1.1.1
The scripts can be run separately or through the snakemake (version 6.10.0) workflow manager.
The full data collection and analysis pipeline is available at https://github.com/MiqG/Zadra_2021.

Others:
-ImageJ 2.3.0
-Prism6

For manuscripts utilizing custom algorithms or software that are central to the research but not yet described in published literature, software must be made available to editors and reviewers. We strongly encourage code deposition in a community repository (e.g. GitHub). See the Nature Portfolio guidelines for submitting code & software for further information.

Data

Policy information about availability of data

All manuscripts must include a data availability statement. This statement should provide the following information, where applicable:

- Accession codes, unique identifiers, or web links for publicly available datasets
- A description of any restrictions on data availability
- For clinical datasets or third party data, please ensure that the statement adheres to our policy

All relevant data supporting the key findings of this study are available within the article, Supplementary Information and Source Data. Any material generated for this study will be shared upon reasonable request within the limits of respective material transfer agreements for as long as they are available in the laboratory. Pan-cancer normalized gene expression, copy number variation and somatic mutations, aneuploidy scores and gene length were obtained as in the TCGA data in the Methods section. Phylogeny data were retrieved from ensambl as in the Sequence sampling in the Methods section. Source data are provided with this paper.

Field-specific reporting

Please select the one below that is the best fit for your research. If you are not sure, read the appropriate sections before making your selection.

☒ Life sciences ☐ Behavioural & social sciences ☐ Ecological, evolutionary & environmental sciences

For a reference copy of the document with all sections, see nature.com/documents/nr-reporting-summary-flat.pdf

Life sciences study design

All studies must disclose on these points even when the disclosure is negative.

Sample size	The sample size was determined in the cell experiment according to the suitable measurable cells found in the cover in every independent experiment. In the experiments with zebrafish embryos, the sample size was determined according to the availability of laid eggs or limited by the experimental settings in live image experiments to maximize sample size with time and space resolution. The sample size in bioinformatics analysis was limited only by the data available on the TCGA database.
Data exclusions	No exclusion of data was performed in any experiment.
Replication	The experiments were always independently replicated at least 3 times replicating the experiment in different days and different cell aliquots. No problems in reproducibility were detected across the experiments presented in the manuscript.
Randomization	In this study sample and organism randomization was not relevant since cofounders are unknown (e.g. zebrafish embryos' genetic background or sex and same genetic background for cells).
Blinding	Investigators were blinded in four experiments presented in the manuscript to avoid biased analysis. These experiments are the ones showed in image 1e, 5b, 5c, 5d. Blinding was not needed in other experiments since the data where automatically processed.

Reporting for specific materials, systems and methods

We require information from authors about some types of materials, experimental systems and methods used in many studies. Here, indicate whether each material, system or method listed is relevant to your study. If you are not sure if a list item applies to your research, read the appropriate section before selecting a response.

Materials & experimental systems

n/a	Involved in the study	
☐	☒	Antibodies
☐	☒	Eukaryotic cell lines
☒	☐	Palaeontology and archaeology
☐	☒	Animals and other organisms
☒	☐	Human research participants
☒	☐	Clinical data
☒	☐	Dual use research of concern

Methods

n/a	Involved in the study	
☒	☐	ChIP-seq
☒	☐	Flow cytometry
☒	☐	MRI-based neuroimaging

Antibodies

Antibodies used	polyE (anti-polyglutamylation), made in-house; DM1A, Sigma T9026; GFP, made in-house; β-tubulin, Abcam ab6046; GT335, Enzo 804-885; CREST, Antibodies Incorporated 15-235; Hec1, Genentex GTX70268; Anti-detyrosinated tubulin, Sigma-Aldrich AB3201; YL1/2, Sigma-Aldrich MAB1864; Ac, Sigma-Aldrich T7451.<br>Secondary Ab: Goat anti-Rabbit IgG, Alexa Fluor™ 488, Invitrogen, A-11008; Goat anti-Rabbit IgG, Alexa Fluor™ 565, Invitrogen, A32732; Goat anti-Mouse IgG, Alexa Fluor™ 488, Invitrogen, A-11001; Goat anti-Mouse IgG, Alexa Fluor™ 488, Invitrogen, A-32727.
Validation	polyE, made in-house, to produce the polyE polyclonal antibody rabbit were injected with a 9 glutamate peptide stretch conjugated with KSL protein, specificity was tested on telokin proteins with c-terminal glutamates of different extend; DM1A, Sigma T9026, the antibody is specific for α-tubulin in immunoblotting assays and may be used for localization of α-tubulin in cultured cells or tissue sections. The antibody reacts best with chicken fibroblasts; GFP, made in-house; the anti-GFP polyclonal antibody was produce in rabbits by injecting the purified GFP protein; β-tubulin, Abcam ab6046; Rabbit polyclonal to beta-Tubulin, suitable for: WB, ICC/IF, IHC-P, IP. Mouse anti-β-tubulin antibody reacts specifically with β tubulin, types I, II, III, and IV of bovine, rat, mouse and human.Anti-Tubulin βIII antibody has been used in western blot analysis. Monoclonal Anti-β-Tubulin antibody produced in mouse has been used in western blotting and immunofluorescence staining; GT335; Enzo 804-885; monoclonal antibody against glutamylated tubulin; CREST, Antibodies Incorporated 15-235; "Serum obtained from an autoimmune patient was purified by protein A chromatography and then tested at a series of dilutions by immunocytochemistry on ethanol-fixed Hep2 cells that were in log-phase growth. The staining pattern obtained was consistent with the pattern expected for anti-centromere staining." Hec1, Genentex GTX70268; reference list in the website page of Genetex. Anti-detyrosinated tubulin, Sigma-Aldrich AB3201; Purified rabbit polyclonal in buffer containing liquid PBS, anti-Tubulin Antibody, Detyrosinated is an antibody against Tubulin for use in IC, IH(P) and WB. YL1/2, Sigma-Aldrich MAB1864Detect Tubulin using this Anti-Tubulin Antibody, clone YL1/2 validated for use in ELISA, IH, IP, RIA & WB;The epitope recognizes a linear sequence requiring an aromatic residue at the C-terminus, with the two adjacent amino acids being negatively charged (represented by Gly-Gly-Tyr in Tyr-Tubulin). Sigma-Aldrich T7451; Monoclonal Anti-Acetylated Tubulin antibody produced in mouse has also been used in quantitative dot blot, immunofluorescence, Western blot, immunocytochemistry, enzyme linked immunosorbent assay (ELISA), solid phase radioimmunoassay (RIA) and electron microscopy.Monoclonal Anti-Acetylated Tubulin antibody produced in mouse has been used in western blot and immunohistochemistry.

Eukaryotic cell lines

Policy information about cell lines

Cell line source(s)	HeLa (ATCC CCL2); stable HeLa cell lines expressing H2B–mRFP/α-tubulin-GFP; H2B–mRFP/PA-α-tubulin–GFP (gift from Patrick Meraldi, University of Geneva); human RPE1; HT-29; HCT-116; MDA-MB-231; MDA-MB-468 and U2O2 cells were obtained from American Type Culture Collection (ATCC).
Authentication	Cells were solely evaluated based on morphological inspection.
Mycoplasma contamination	Mycoplasma testing is conducted routinely on the cell lines used. No contamination in the Cell line batches presented in the manuscript was ever detected.
Commonly misidentified lines<br>(See ICLAC register)	No commonly misidentified lines have been used in this study.

Animals and other organisms

Policy information about studies involving animals; ARRIVE guidelines recommended for reporting animal research

Laboratory animals	The following Zebrafish (Danio rerio) strains were used in this study: AB wild-type zebrafish and the transgenic fish line Tg(actb2:h2a-mCherry) (Zfin: e103Tg, https://zfin.org/ZDB-ALT-111207-6#summary). Zebrafish adults (male-female) from the respective lines were crossed between 5 to 12 months of age to obtain fertilized eggs.<br>Embryos were analyzed between 4 hpf (Hour post fertilization) to 36 hpf according to the experiment. The sex of the embryos was unknown since no dimorphism is present at such early stages.
Wild animals	No wild animals were used in this project.
Field-collected samples	No field- collected samples were used in this project.
Ethics oversight	All protocols using zebrafish conformed to the guidelines from the European Community Directive and Spanish legislation for the experimental use of animals and were approved by the Institutional Animal Care and Ethics Committee (PRBB–IACUEC). Experiments were carried out in accordance with the principles of the 3Rs.

Note that full information on the approval of the study protocol must also be provided in the manuscript.
